# Replication Stress Tolerance in Adult T-Cell Leukemia

**DOI:** 10.3390/biom16091310

**Published:** 2026-09-09

**Authors:** Yusuke Okamoto, Masayuki Kobayashi, Takashi Sakamoto, Kotaro Shirakawa, Akifumi Takaori-Kondo

**Affiliations:** 1 Department of Hematology, Graduate School of Medicine, Kyoto University, Kyoto 606-8303, Japan; okamotoy@kuhp.kyoto-u.ac.jp (Y.O.); mkobayas@kuhp.kyoto-u.ac.jp (M.K.); tsakamo@kuhp.kyoto-u.ac.jp (T.S.); kotash@kuhp.kyoto-u.ac.jp (K.S.); 2 Department of Hematology, Japan Baptist Hospital, Kyoto 606-8273, Japan

**Keywords:** replication stress, ATL, HTLV1, TDP1, abacavir, SLFN11

## Abstract

Replication stress (RS) represents a major vulnerability of cancer cells treated with nucleoside analogs and related antimetabolites; however, tumors frequently acquire tolerance mechanisms that permit survival despite persistent DNA lesions. This review examines molecular determinants of RS tolerance, focusing on human T-cell leukemia virus type 1 (HTLV-1)-mediated adult T-cell leukemia/lymphoma (ATL) as a model of virus-mediated rewiring of DNA damage responses. Chain-terminating nucleoside analogs generate aberrant replication intermediates, including blocked 3’ DNA termini, mis-incorporated bases, and stalled replication forks. In ATL, viral oncoproteins suppress key components of replication stress response pathways, notably tyrosyl-DNA phosphodiesterase 1 (TDP1) and mismatch repair (MMR), thereby creating exploitable repair deficiencies. Consistent with this vulnerability, ATL cells exhibit marked sensitivity to replication stress–inducing agents such as irinotecan (CPT-11) and the chain-terminating nucleoside analog abacavir. Recent CRISPR-based functional genomics studies further identify Schlafen 11 (SLFN11) as an independent and dominant regulator of RS sensitivity. SLFN11 determines the fate of stressed replication forks independently of lesion processing, acting as an execution factor that converts otherwise tolerable RS into irreversible replication arrest. We conclude by discussing therapeutic strategies that exploit RS tolerance defects in ATL, including biomarker-guided nucleoside analog therapy, and rational combination approaches targeting compensatory RS pathways.

## 1. Introduction

Faithful duplication of the genome during S phase is essential for maintaining genomic integrity and cellular viability. DNA replication, however, is continuously challenged by endogenous and exogenous obstacles that slow or stall replication fork progression, collectively referred to as replication stress (RS). Endogenous sources of RS include oncogene activation, transcription–replication conflicts, DNA secondary structures, oxidative DNA damage, and depletion of nucleotide pools, whereas exogenous sources include radiation and numerous chemotherapeutic agents that directly interfere with DNA synthesis or replication fork progression [1]. Although transient replication stress is a physiological consequence of normal cell proliferation and can usually be resolved through checkpoint activation and DNA repair, persistent or excessive RS results in replication fork collapse, chromosome breakage, and genomic instability, all of which contribute to tumor initiation and progression [2]. Indeed, elevated RS is now recognized as a hallmark of many human cancers and represents one of the most therapeutically exploitable vulnerabilities of malignant cells.

The ability of cancer cells to survive chronic RS depends not only on canonical DNA repair pathways but also on a broad network of replication stress tolerance mechanisms that stabilize stalled replication forks, facilitate lesion bypass, and coordinate replication restart. These adaptive responses enable cancer cells to proliferate despite continuous DNA damage and replication obstacles. Consequently, many anticancer therapies aim not simply to induce DNA damage but to overwhelm or disable these tolerance mechanisms, thereby converting manageable replication perturbations into irreversible replication failure and cell death [2]. Understanding the molecular basis of RS tolerance has therefore become increasingly important for the development of mechanism-based therapeutic strategies.

Among DNA-targeting agents, nucleoside analogs represent one of the most clinically important classes of RS-inducing drugs. These compounds are widely used for the treatment of hematologic malignancies, solid tumors, and viral infections because they closely resemble physiological nucleosides and are readily incorporated into newly synthesized DNA. Depending on their chemical structures, nucleoside analogs interfere with DNA replication through several distinct mechanisms. Chain-terminating analogs lack a functional 3′-hydroxyl group required for phosphodiester bond formation, resulting in immediate termination of DNA synthesis. Other analogs induce abnormal base pairing, alter DNA helical structure, or interfere with DNA polymerase progression without causing complete chain termination. Consequently, nucleoside analog incorporation generates a diverse spectrum of DNA lesions, including chemically blocked 3′ termini, mis-incorporated nucleotides, persistent single-stranded DNA gaps, stalled replication forks, and secondary DNA double-strand breaks arising from replication fork collapse [3,4,5]. The nature of these lesions ultimately determines which DNA repair pathways are engaged and whether cells successfully tolerate or succumb to replication stress.

Cellular responses to nucleoside analog-induced DNA damage are remarkably heterogeneous. Some lesions are efficiently removed through specialized DNA end-processing enzymes or bypassed by translesion synthesis (TLS) polymerases, allowing DNA replication to resume with minimal consequences. Other lesions activate checkpoint signaling through the ATR–CHK1 pathway, induce replication fork reversal, or stimulate homologous recombination-mediated fork protection. In contrast, lesions that cannot be adequately processed eventually trigger replication catastrophe, chromosome fragmentation, and apoptosis. Thus, the biological outcome of nucleoside analog treatment is determined not simply by the extent of DNA damage but by the dynamic balance between mechanisms that promote lesion resolution and those that enforce irreversible replication arrest [3,4,5].

One of the key enzymes involved in processing replication-associated DNA lesions is tyrosyl-DNA phosphodiesterase 1 (TDP1). Initially characterized for its ability to hydrolyze the covalent phosphotyrosyl linkage between topoisomerase I (Top1) and DNA, TDP1 is now recognized as a versatile DNA end-processing enzyme capable of removing diverse 3′-blocking lesions generated during abortive repair reactions, oxidative damage, and incorporation of certain chain-terminating nucleoside analogs [4]. By restoring a functional 3′-hydroxyl terminus, TDP1 facilitates repair synthesis and replication fork restart. In parallel, the mismatch repair (MMR) pathway contributes to cellular responses to base-modifying nucleoside analogs by recognizing abnormal base pairs and activating DNA damage signaling. Rather than functioning solely as an error-correction system, MMR can convert relatively innocuous nucleotide misincorporation into highly cytotoxic replication stress through repeated cycles of excision and checkpoint activation. Together with TLS polymerases, homologous recombination proteins, and replication fork remodeling factors, TDP1 and MMR constitute major determinants of replication stress tolerance.

Adult T-cell leukemia/lymphoma (ATL), an aggressive mature T-cell malignancy caused by persistent infection with human T-cell leukemia virus type 1 (HTLV-1), provides a unique disease model for investigating how replication stress tolerance can be rewired during oncogenesis. Unlike many sporadic cancers in which DNA repair defects arise through somatic mutations, ATL develops in the context of chronic viral infection, allowing viral proteins to directly manipulate host DNA damage response pathways. HTLV-1 regulatory proteins, particularly HTLV-1 bZIP factor (HBZ) and Tax, promote leukemogenesis through multiple mechanisms, including dysregulation of cell-cycle progression, activation of proliferative signaling pathways, induction of oxidative stress, and impairment of genome maintenance systems [6,7,8]. These viral activities create an environment characterized by persistent replication stress and progressive genomic instability, both of which are essential for malignant transformation and clonal evolution. In addition to impairing DNA repair pathways, HTLV-1-mediated oncogenic signaling may further exacerbate replication stress through increased oxidative stress, transcriptional hyperactivation, metabolic rewiring, and deregulated replication origin firing. Increased cellular proliferation and transcriptional activity can enhance transcription–replication conflicts and increase the demand on replication machinery, while metabolic alterations may affect nucleotide availability and the capacity to sustain DNA synthesis. In particular, Tax-mediated activation of NF-κB has been shown to promote transcriptional hyperactivation and accumulation of R-loops, which can generate transcription–replication conflicts and interfere with replication fork progression. Persistent transcription–replication conflicts may promote replication fork stalling and collapse, resulting in the accumulation of DNA strand breaks and further contributing to genomic instability in HTLV-1-infected and ATL cells [9,10]. Collectively, these endogenous sources of replication stress are likely to cooperate with impaired DNA damage responses to establish a chronic replication stress environment in ATL.

Recent studies have demonstrated that HTLV-1 selectively suppresses key components of the host DNA damage response. HBZ inhibits the transcription factor NRF1, resulting in reduced expression of TDP1 and impaired processing of blocked 3′ DNA termini [11]. Independently, HTLV-1 viral factors attenuate the mismatch repair machinery, thereby altering DNA damage sensing while simultaneously increasing mutation accumulation [12]. These findings suggest that viral-mediated DNA repair deficiency is not merely a byproduct of malignant transformation but rather an active strategy that promotes genomic diversity while creating unique therapeutic vulnerabilities. Indeed, the selective sensitivity of TDP1-deficient ATL cells to the chain-terminating nucleoside analog abacavir represents one of the first examples of exploiting a virus-induced DNA repair defect through synthetic lethality [11].

**Figure 1:**
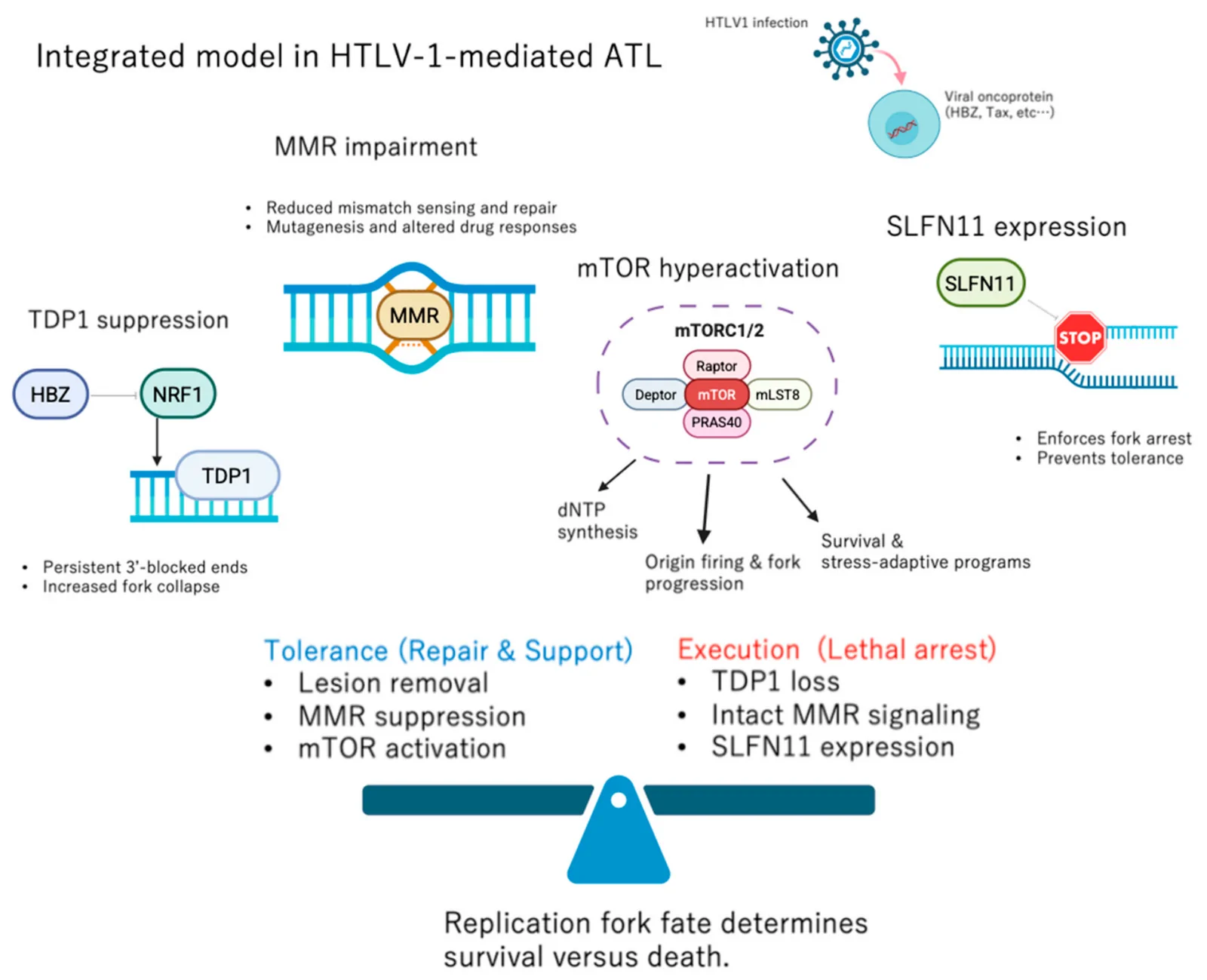
Integrated model of replication stress tolerance in HTLV-1-mediated ATL. HTLV-1-induced suppression of TDP1 and mismatch repair, together with mTOR hyperactivation and backup tolerance pathways, promotes survival under conditions of replication stress. In contrast, SLFN11 functions as a replication stress execution factor that prevents fork restart and converts replication stress into irreversible fork collapse and cell death. The balance between these opposing processes determines therapeutic responses and represents a framework for biomarker-guided interventions in ATL. This figure is created with BioRender.com.

More recently, the discovery of Schlafen 11 (SLFN11) has fundamentally changed current concepts of replication stress biology. Initially identified through unbiased pharmacogenomic analyses as one of the strongest predictors of sensitivity to DNA-damaging agents, SLFN11 was subsequently shown to function as a replication stress execution factor rather than a DNA repair enzyme [13,14,15,16]. Upon replication stress, SLFN11 is recruited to stalled replication forks, where it suppresses replication helicase progression, inhibits dormant origin firing, and prevents replication restart. Consequently, SLFN11 converts otherwise repairable replication stress into irreversible replication arrest and cell death. Importantly, genome-wide CRISPR screening performed in TDP1-deficient cells demonstrated that loss of SLFN11 confers resistance to topoisomerase I inhibitors independently of TDP1-mediated lesion processing, indicating that lesion resolution and replication execution represent genetically distinct determinants of therapeutic response [17]. These observations have shifted the field from viewing DNA repair as the sole determinant of drug sensitivity toward a broader model in which the ultimate fate of replication forks depends on the balance between damage resolution and damage execution (Figure 1).

**Figure 2:**
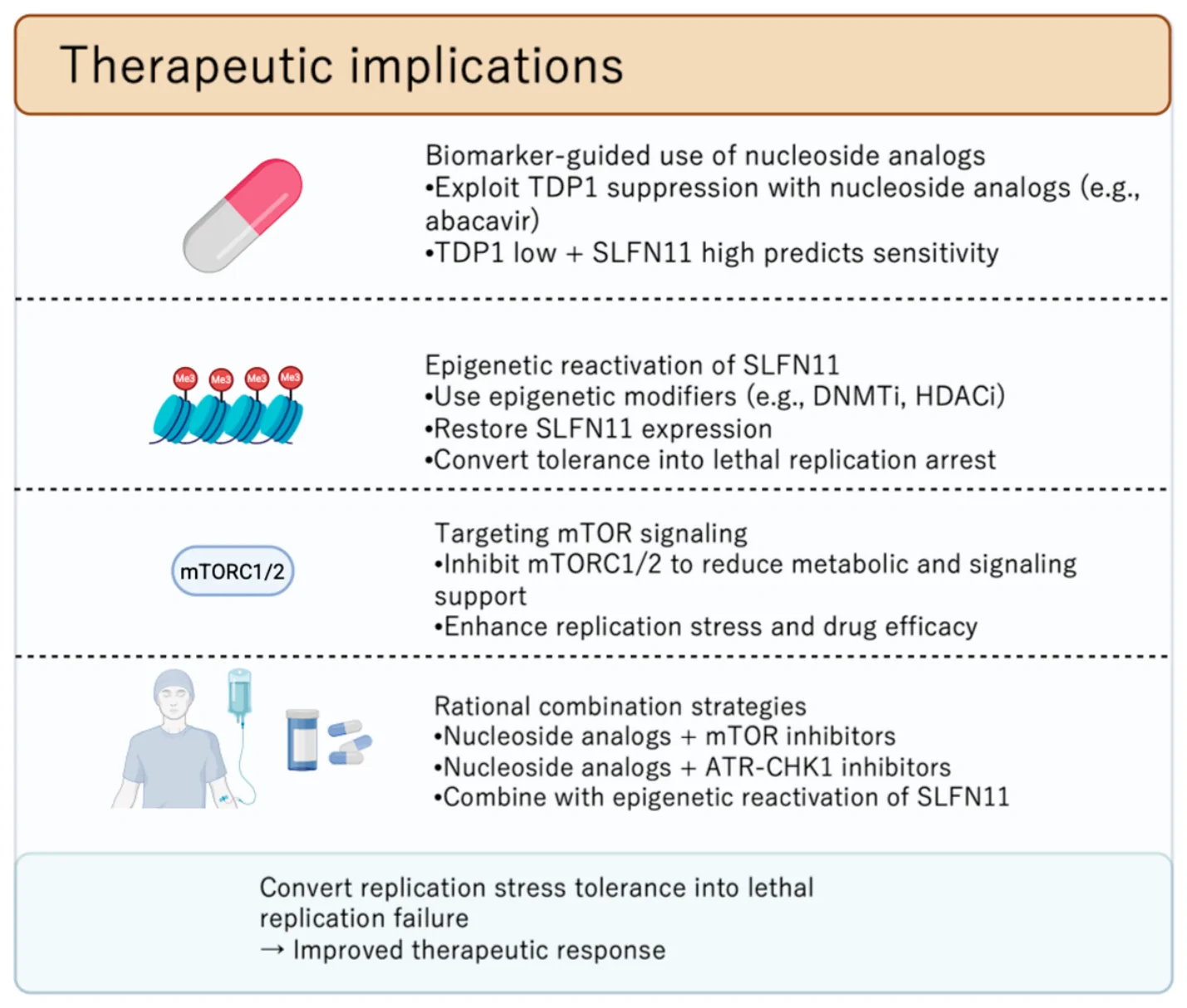
Therapeutic implications of replication stress-targeted therapy in HTLV-1-mediated ATL. Viral suppression of TDP1 and modulation of SLFN11 create therapeutic vulnerabilities that can be exploited using nucleoside analogs, such as abacavir. Combined assessment of TDP1 and SLFN11 may serve as predictive biomarkers for treatment response. Epigenetic reactivation of SLFN11, together with mTOR or ATR–CHK1 inhibition, is expected to enhance replication stress, prevent fork recovery, and convert tolerated DNA damage into irreversible replication failure, thereby improving therapeutic efficacy. This figure is created with BioRender.com.

In this review, we summarize current understanding of replication stress tolerance mechanisms induced by nucleoside analogs and related DNA-targeting agents, with particular emphasis on virus-associated DNA repair rewiring in HTLV-1-mediated ATL. We discuss how suppression of TDP1 and mismatch repair, together with modulation of metabolic signaling pathways and the emerging role of SLFN11, collectively determine whether replication stress is tolerated or converted into lethal replication failure [6,7,8,11,12,18]. Finally, we propose an integrated conceptual framework in which therapeutic responses are governed by the balance between lesion processing and replication execution (Figure 1), and discuss how this model may facilitate biomarker-guided patient stratification and the development of rational combination therapies targeting replication stress across diverse malignancies (Figure 2).

## 2. Nucleoside Analogs and the Molecular Lesions They Create

### 2.1. Incorporation of Nucleoside Analogs into Nascent DNA

Chain-terminating nucleoside analogs (CTNAs) and other antimetabolites are incorporated into nascent DNA strands by replicative DNA polymerases due to their structural similarity to natural nucleosides [19]. However, many CTNAs lack a functional 3’-hydroxyl group or contain chemical modifications at the sugar or base moiety, preventing further phosphodiester bond formation [19]. As a result, DNA synthesis is abruptly halted at the site of incorporation. This immediate chain termination generates abnormal DNA ends that are intrinsically incompatible with canonical extension and require specialized processing for replication to resume.

### 2.2. Aberrant 3′ Termini and the Need for End-Processing Enzymes

The abnormal 3’ termini generated by CTNA incorporation or abortive repair reactions represent a critical molecular lesion underlying replication stress [3,4,5]. These termini may include 3’-phosphates, 3’-phosphoglycolates, or covalent protein–DNA adducts formed indirectly through topoisomerase I trapping. Enzymes such as TDP1 play a central role in resolving these blocked ends, thereby restoring a ligatable or extendable 3’-OH(3–5). In the absence of efficient end processing, stalled replication forks persist and become prone to collapse, leading to cytotoxic DNA double-strand breaks.

### 2.3. Base Misincorporation and MMR–Dependent Responses

In addition to chain termination, certain nucleoside analogs are fully incorporated into DNA but introduce subtle base-pairing abnormalities. These mis-pairs are detected by the MMR machinery, such as MSH2 during or shortly after replication. MMR engagement at sites containing nucleoside analogs can have dual outcomes: productive repair that removes the analog-containing strand, or repeated futile repair cycles that amplify single-stranded DNA regions and activate checkpoint signaling [20]. Thus, MMR status critically influences whether analog incorporation results in tolerance, mutagenesis, or cell death.

### 2.4. Single-Strand Breaks Arising from Excision and Repair Attempts

**Figure 3:**
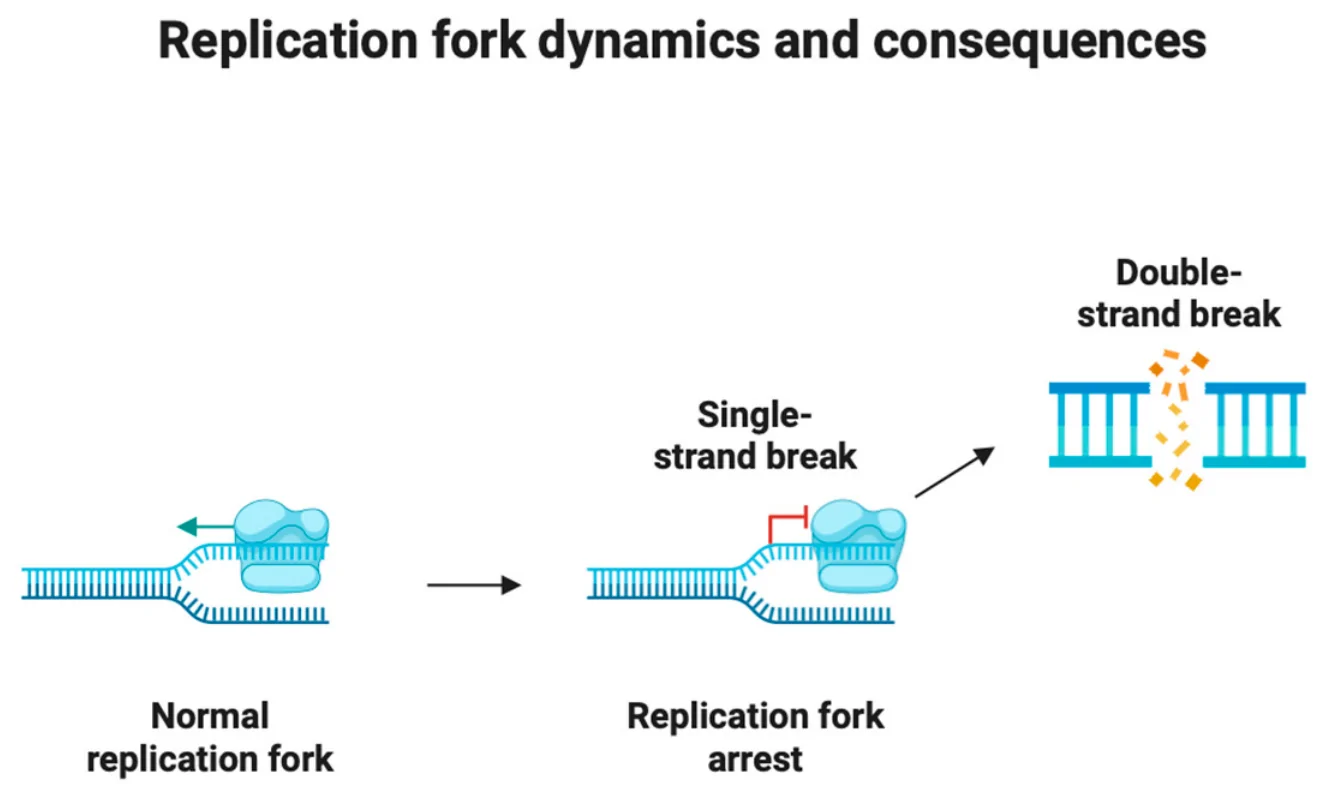
Replication fork dynamics and consequences. Under normal conditions, replication forks progress continuously along the DNA template to ensure faithful genome duplication. When a replication fork encounters a DNA lesion, such as a single-strand break or a blocked 3′ DNA end, DNA synthesis is impeded, resulting in replication fork arrest. If the lesion is not efficiently resolved, prolonged fork stalling can lead to replication fork collapse and the generation of highly cytotoxic one-ended DNA double-strand breaks. This figure is created with BioRender.com.

Processing of nucleoside analog-containing DNA frequently involves excision-based pathways, including base excision repair (BER) and MMR-associated exonuclease activity. These reactions transiently generate single-strand breaks (SSBs) and gaps. Under conditions of high replication stress or defective coordination of repair, such SSBs can be converted into one-ended double-strand breaks when encountered by advancing replication forks (Figure 3). Therefore, lesions initially confined to a single-strand can escalate into highly toxic structures through replication-coupled mechanisms [21].

### 2.5. Replication Stress Tolerance Pathways at Stalled Forks

Cellular tolerance to nucleoside analog-induced lesions relies on multiple, partially redundant pathways that stabilize and restart stalled replication forks. These include: (i) removal of aberrant DNA termini by enzymes such as TDP1; (ii) gap filling or lesion bypass mediated by translesion synthesis (TLS) polymerases, which allow replication to continue at the cost of increased mutagenesis; and (iii) coordination of fork restart and protection through fork reversal and homologous recombination–related factors, including RAD51 [3,4,5]. Upon persistent replication stress, stalled forks can undergo reversal, in which the nascent DNA strands anneal to form a four-way junction-like structure that temporarily limits further fork progression and provides an opportunity for lesion processing and repair [22,23]. These reversed forks must then be protected from excessive nucleolytic degradation, particularly through RAD51- and BRCA-dependent mechanisms, to preserve replication competence. If fork protection is compromised, nucleases such as MRE11, DNA2, and EXO1 can promote degradation of nascent DNA, resulting in fork destabilization and increased genome instability [23,24,25]. Conversely, properly protected reversed forks can be processed and subsequently restarted through homologous recombination–dependent or replication restart mechanisms [22,23]. In ATL, where persistent endogenous replication stress is combined with viral-mediated impairment of DNA repair pathways, the ability to protect and restart stalled or reversed forks is likely to become particularly important for maintaining cell viability. Thus, alterations in fork reversal, fork protection, nucleolytic processing, and restart may represent additional determinants of replication stress tolerance and potential therapeutic vulnerabilities in ATL. The balance and efficiency of these pathways determine whether nucleoside analog-induced replication stress is resolved, tolerated, or converted into lethal genomic instability.

## 3. TDP1 Suppression in HTLV 1-Associated ATL: Mechanistic and Therapeutic Consequences

### 3.1. Biological Role of TDP1 at Stalled Replication Forks

TDP1 is a key DNA end-processing enzyme that resolves a variety of obstructive 3’ DNA termini. Its canonical role is the hydrolysis of covalent 3’-phosphotyrosyl bonds formed by trapped topoisomerase I cleavage complexes, but TDP1 also processes diverse abnormal 3’ adducts generated during base excision repair, abortive ligation, or incorporation of chain-terminating nucleoside analogs. At stalled replication forks, these blocked termini represent critical barriers to fork restart. Efficient TDP1 activity restores a ligatable or extendable 3’-hydroxyl group, thereby permitting repair synthesis and continuation of DNA replication [3,4,5].

In the absence of functional TDP1, unprocessed 3’-blocked termini accumulate and persist at sites of replication stress. Such lesions impede fork restart, increase reliance on backup tolerance pathways, and predispose forks to collapse into one-ended double-strand breaks, creating a state of heightened vulnerability to replication stress–inducing agents.

### 3.2. TDP1 Deficiency as a Recurrent Feature of HTLV 1-Associated ATL

Key studies have demonstrated that TDP1 deficiency or suppression is not incidental but rather a recurring molecular feature in adult T-cell leukemia (ATL). Our group first noted that a subset of ATL cells exhibits markedly reduced TDP1 expression and activity, distinguishing them from normal T cells and other hematologic malignancies [11]. This deficiency establishes a unique repair landscape in which specific classes of DNA lesions—particularly those involving abnormal 3’ termini—cannot be efficiently resolved.

Subsequently, we elucidated a viral mechanism underlying this phenotype by identifying the HTLV-1 bZIP factor (HBZ) as a suppressor of TDP1 transcription [18]. HBZ inhibits the transcription factor NRF1, which is required for basal TDP1 expression, thereby directly linking viral oncogenic activity to repression of a defined DNA repair enzyme. This finding provides a mechanistic explanation for why TDP1 suppression is selectively observed in HTLV-1-mediated ATL rather than being a generic consequence of transformation [6,7,8,18].

### 3.3. Exploiting TDP1 Suppression with Nucleoside Analogs

**Figure 4:**
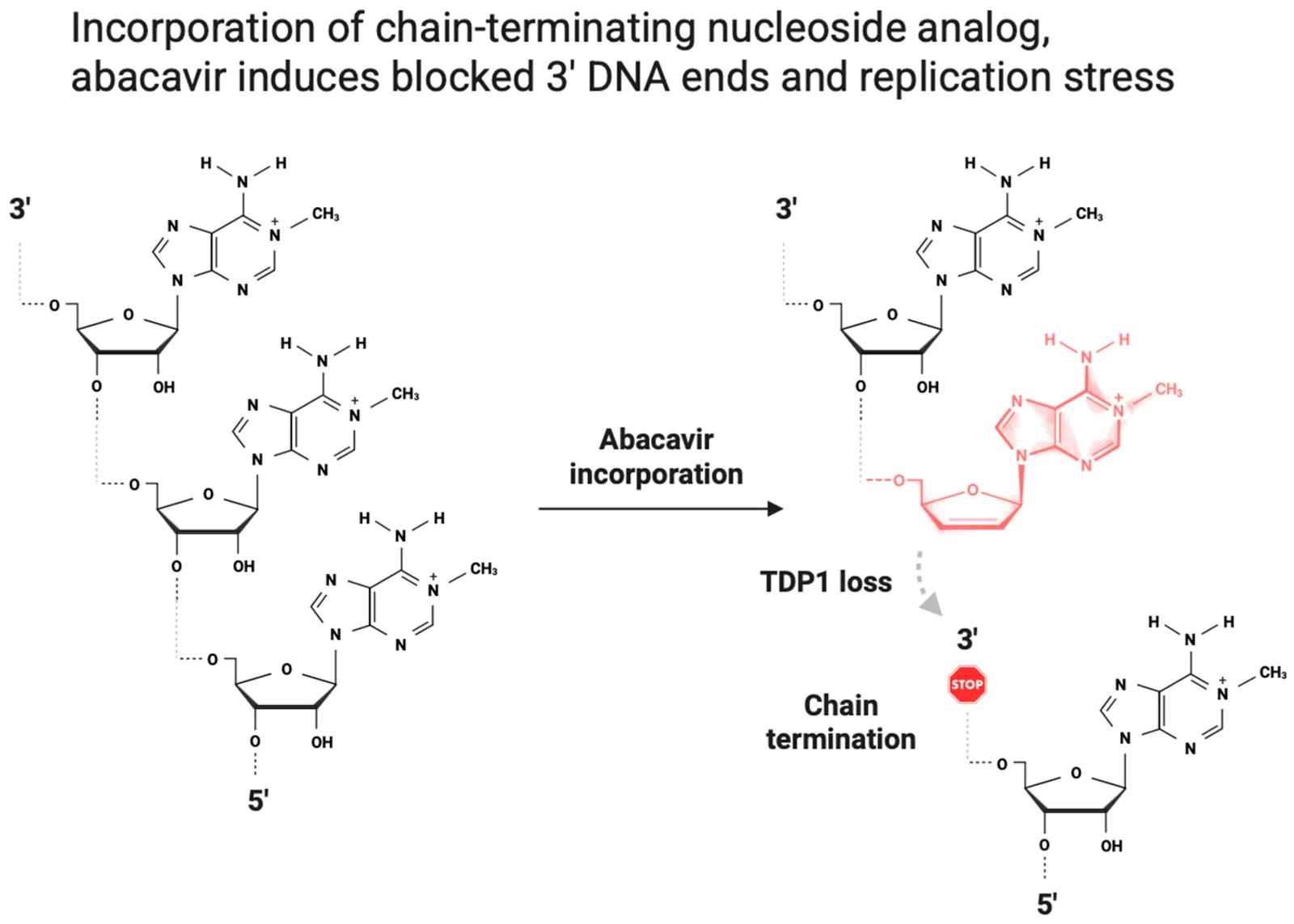
Incorporation of chain-terminating nucleoside analog, abacavir induces blocked 3’ DNA ends and replication stress. Unlike natural nucleotides, abacavir lacks a functional 3′-hydroxyl (3′-OH) group required for phosphodiester bond formation, thereby preventing the addition of subsequent nucleotides and terminating DNA chain elongation. Unrepaired blocked DNA ends stall replication fork progression, leading to replication stress and persistent fork arrest. Efficient removal of blocked 3′ termini by tyrosyl-DNA phosphodiesterase 1 (TDP1) promotes replication fork restart and cell survival, whereas failure to resolve these lesions results in prolonged fork stalling, replication fork collapse, DNA double-strand break formation, and ultimately cell death. This figure is created with BioRender.com.

The therapeutic implications of TDP1 suppression were elegantly demonstrated by our group using a chemical–genetic screening approach [11]. They identified the nucleoside analog abacavir, an HIV reverse transcriptase inhibitor, as a compound that selectively induces cytotoxicity in TDP1-deficient ATL cells. Unlike many nucleoside analogs that primarily induce base mispairing or inhibit DNA polymerase progression, abacavir acts as a chain-terminating nucleoside analog that generates blocked 3′ DNA termini following incorporation into nascent DNA. These lesions require specialized end-processing enzymes, particularly TDP1, for restoration of a functional 3′-hydroxyl terminus. Consequently, TDP1-deficient ATL cells are unable to efficiently resolve abacavir-induced lesions, providing a mechanistic basis for the selective synthetic-lethal interaction between abacavir and TDP1 suppression (Figure 4).

Importantly, abacavir exhibits minimal toxicity in TDP1-proficient cells, underscoring the specificity of this vulnerability. This selectivity exemplifies a synthetic-lethal interaction in which loss of a DNA repair function unmasks sensitivity to a drug that would otherwise be tolerated.

### 3.4. TDP1 Loss as a Paradigmatic Targetable Vulnerability in ATL

Collectively, these findings establish the HBZ–NRF1–TDP1 axis as a paradigmatic example of virus-induced DNA repair deficiency that can be therapeutically exploited [11,12,18]. TDP1 suppression sensitizes ATL cells to nucleoside analogs and potentially to other agents that generate blocked 3’ termini, including topoisomerase I poisons. More broadly, this model illustrates how oncogenic viruses can rewire replication stress responses to favor leukemogenesis while simultaneously creating highly specific therapeutic liabilities.

In the context of emerging replication stress-targeted therapies, TDP1-deficient ATL provides a compelling framework for biomarker-driven treatment strategies and rational combination approaches, particularly when integrated with additional tolerance regulators such as SLFN11 and ATR–CHK1 signaling [13,14,15,16].

## 4. MMR Impairment by Viral Oncoproteins and Effects on Nucleoside Ana-Log Response

### 4.1. Canonical Roles of MMR in Replication Fidelity and Damage Signaling

**Figure 5:**
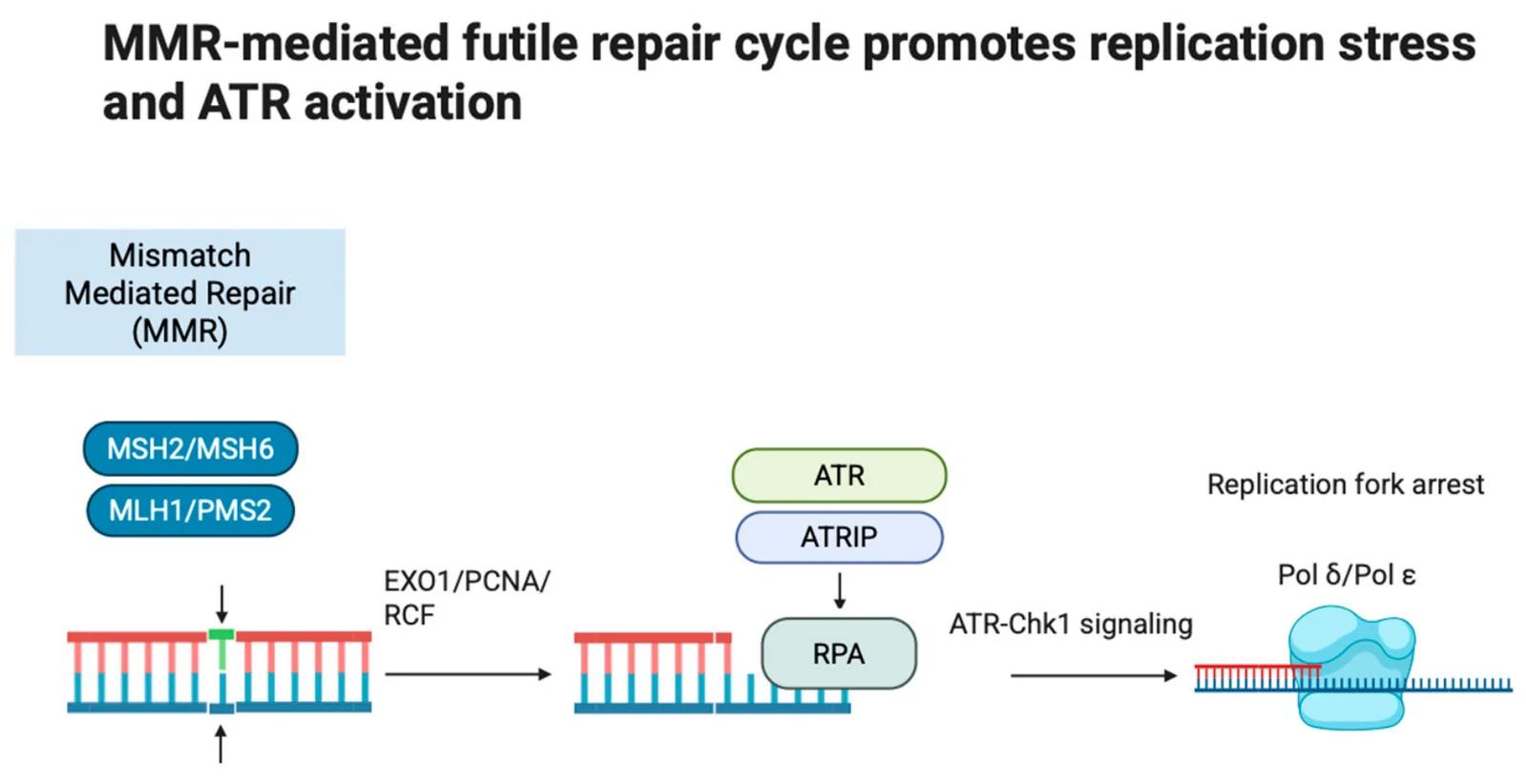
MMR-mediated futile repair cycle promotes replication stress and ATR activation. Following DNA replication, incorporated nucleoside analogs or chemically modified nucleotides are recognized as aberrant bases by the mismatch repair (MMR) machinery. The MutSα complex (MSH2–MSH6) detects the abnormal nucleotide and recruits the MutLα complex (MLH1–PMS2), which initiates excision of the newly synthesized DNA strand through exonuclease 1 (EXO1). Because the modified nucleotide remains present in the template or is repeatedly reinserted during DNA resynthesis, MMR repeatedly recognizes the lesion and initiates successive rounds of excision and repair without removing the underlying damage, a process referred to as futile repair. Recurrent excision generates persistent single-stranded DNA (ssDNA), which becomes coated by replication protein A (RPA), leading to activation of the ATR–CHK1 replication stress signaling pathway. Sustained ATR signaling promotes prolonged replication fork arrest and, when damage persists, replication fork collapse, DNA double-strand break (DSB) formation, and cell death. This figure is created with BioRender.com.

The MMR system is a core genome maintenance pathway that detects and repairs base–base mismatches and small insertion–deletion loops generated during DNA replication [12,21,26]. Beyond its role in maintaining replication fidelity, MMR also functions as a damage-sensing and signaling pathway. When MMR proteins encounter certain noncanonical bases or chemically modified nucleotides that cannot be efficiently repaired, repeated cycles of excision and resynthesis (“futile repair”) occur (Figure 5). These cycles generate persistent single-stranded DNA regions, activate ATR-dependent checkpoint signaling, and ultimately convert otherwise tolerated lesions into cytotoxic replication stress [27].

This signaling function of MMR is particularly relevant for nucleoside analogs that are incorporated into DNA without causing immediate chain termination but instead perturb base pairing or DNA geometry. In such contexts, intact MMR is required to translate analog incorporation into an effective antitumor response.

### 4.2. Viral-Mediated Suppression of MMR in HTLV-1-Associated ATL

We demonstrated that HTLV-1 viral factors impair key components of the MMR machinery in ATL through transcriptional and regulatory mechanisms rather than by inducing mutations in the corresponding genes [12]. This viral-mediated suppression represents a targeted rewiring of host genome surveillance rather than a passive consequence of malignant transformation. By attenuating MMR activity, HTLV-1–infected cells can tolerate a higher burden of replication errors and DNA base damage, thereby increasing baseline genomic instability and accelerating leukemogenic evolution [6,7,8,12].

Importantly, MMR impairment in ATL is often partial rather than complete, suggesting a selective advantage: sufficient loss of function to evade damage signaling and cell-cycle arrest, while retaining minimal repair capacity compatible with continued proliferation under chronic replication stress.

### 4.3. Consequences of MMR Deficiency for Nucleoside Analog Response

MMR deficiency has dual and context-dependent effects on responses to nucleoside analog therapy [12,20,21,26,28]. On the one hand, loss of MMR abrogates damage signaling in response to base analog-induced mis-pairs, allowing cells to tolerate lesions that would otherwise trigger checkpoint activation and cell death. This mechanism can confer resistance to nucleoside analogs whose cytotoxicity relies on MMR engagement.

On the other hand, MMR deficiency promotes the accumulation of mutations and secondary lesions, which can indirectly exacerbate replication stress and create new dependencies on backup tolerance pathways. Thus, while MMR loss may blunt the immediate cytotoxic effect of certain analogs, it can simultaneously increase vulnerability to agents that induce chain termination, replication fork collapse, or ATR–CHK1 dependency.

### 4.4. Integration of MMR Impairment into the Replication Stress Tolerance Framework

In HTLV-1-mediated ATL, viral suppression of MMR should be viewed as one component of a broader replication stress tolerance network that includes TDP1 suppression, SLFN11 modulation, and mTOR-dependent metabolic support. Within this framework, MMR impairment shifts the cellular response to nucleoside analogs away from signaling-mediated lethality and toward tolerance and mutagenesis.

Understanding which classes of nucleoside analogs require MMR for efficacy, and which act independently through chain termination or blocked termini, is therefore essential for rational drug selection and combination therapy design in ATL.

## 5. Signaling Rewiring (mTOR) and Cooperation with Repair Defects in ATL Therapy

### 5.1. mTOR Signaling as a Modulator of Replication Stress Tolerance

Kawata et al. demonstrated that dual inhibition of mTORC1 and mTORC2 exerts significant antitumor activity in adult T-cell leukemia (ATL), highlighting the mTOR pathway as a critical survival axis in this disease [29]. Although mTOR is not a canonical DNA damage response or DNA repair factor, it plays a central role in regulating cellular processes that directly influence replication stress tolerance [29,30,31,32]. These include control of nucleotide biosynthesis through regulation of ribonucleotide reductase and one-carbon metabolism, modulation of replication origin firing via growth and nutrient sensing, and activation of stress-adaptive transcriptional and translational programs.

Through these mechanisms, hyperactive mTOR signaling enables leukemic cells to buffer replication stress by ensuring sufficient dNTP supply, maintaining replication fork progression, and activating pro-survival responses under conditions of genotoxic stress. In ATL, where intrinsic replication stress is already elevated due to viral oncogenic signaling and repair pathway suppression, mTOR activity may therefore represent a key non-genetic enabler of replication stress tolerance [29,30,31,32].

### 5.2. Functional Interplay Between mTOR Inhibition and TDP1/MMR Defects

In the context of TDP1 suppression and MMR impairment, mTOR inhibition is predicted to shift the balance from stress tolerance toward lethal replication failure. First, suppression of mTOR-driven nucleotide biosynthesis reduces intracellular dNTP pools, exacerbating fork stalling and increasing the likelihood that unrepaired 3’-blocked termini persist in TDP1-deficient cells. Second, inhibition of mTORC1/2 blunts downstream pro-survival and metabolic signaling pathways that normally support recovery from replication stress, including pathways that promote cell-cycle progression and stress adaptation.

Third, and most importantly from a therapeutic perspective, mTOR inhibition may synergize with nucleoside analogs or DNA repair-targeted agents. In TDP1- or MMR-deficient ATL cells, nucleoside analog-induced lesions already challenge replication fork integrity. Concurrent mTOR inhibition deprives cells of the metabolic and signaling resources required to tolerate this stress, thereby converting otherwise sublethal damage into irreversible replication catastrophe [29,30,31,32,33].

### 5.3. Implications for Combination Therapy Design

These mechanistic considerations provide a strong rationale for combination strategies that integrate DNA repair vulnerabilities with metabolic and signaling interventions. Specifically, exploiting a defined repair defect (such as TDP1 deficiency) using a nucleoside analog may be potentiated by simultaneous inhibition of mTOR signaling, which undermines the cellular infrastructure supporting replication stress tolerance. Such combinations are conceptually distinct from traditional cytotoxic regimens, as they aim to selectively disable the adaptive capacity of leukemic cells rather than merely increasing the burden of DNA damage.

In this framework, mTOR inhibitors function as sensitizers of replication stress rather than as standalone cytotoxic agents, positioning them as valuable components of biomarker-driven, mechanism-based therapeutic strategies in ATL [29,30,31,32,33].

## 6. HTLV-1-Mediated Remodeling of DNA Damage Response Pathways

Although TDP1 and MMR represent the principal DNA repair pathways discussed in this review, HTLV-1 has been reported to perturb several additional components of the DNA damage response. Tax can impair base excision repair (BER), in part through transcriptional repression of DNA polymerase β, thereby reducing the capacity to repair oxidative DNA lesions. Tax has also been reported to interfere with homologous recombination (HR), including through NF-κB-dependent suppression of HR activity and sequestration or functional impairment of factors involved in DNA damage signaling and repair, such as BRCA1 and MDC1. In parallel, Tax attenuates DNA damage checkpoint signaling by interfering with ATM/MDC1-dependent signaling and by modulating checkpoint kinases including CHK1 and CHK2, thereby facilitating cell-cycle progression despite the presence of DNA lesions. These alterations, together with reported effects on nucleotide excision repair and non-homologous end joining, may contribute to the accumulation and persistence of replication-associated DNA damage in HTLV-1-infected and ATL cells. Although these pathways likely cooperate to shape the overall DNA damage response in ATL, the present review focuses primarily on TDP1 and MMR because of their more direct relevance to the processing and tolerance of nucleoside analog-induced replication-associated lesions.

### 6.1. Modulation of DNA Repair Pathways

HTLV-1, particularly through the viral oncoprotein Tax, has been reported to perturb multiple additional DNA repair mechanisms. Tax suppresses the expression of DNA polymerase β (POLB), a key component of the BER pathway, thereby impairing the repair of DNA lesions generated by oxidative stress and other endogenous or exogenous insults [34]. Tax also impairs nucleotide excision repair (NER), in part through increased PCNA expression and disruption of the normal coupling between DNA damage recognition and replication control [35]. In addition, Tax-mediated activation of NF-κB has been associated with impaired homologous recombination (HR), potentially compromising the repair of DNA double-strand breaks and replication-associated lesions. Tax has also been reported to interfere with non-homologous end joining (NHEJ), including through altered regulation of DNA-PK and Ku80, further contributing to the persistence of DNA double-strand breaks [36]. These observations indicate that HTLV-1 does not simply inhibit a single DNA repair pathway but broadly remodels the cellular network responsible for maintaining genome integrity. Such coordinated perturbation may be particularly relevant in the setting of chronic replication stress, where simultaneous impairment of multiple repair pathways can increase the likelihood that stalled replication forks and replication-associated lesions progress to persistent DNA damage and genomic instability.

### 6.2. Disruption of DNA Damage Checkpoint Signaling

HTLV-1 also modulates DNA damage sensing and checkpoint signaling, thereby altering the cellular response to replication-associated DNA lesions. Tax interferes with the ATM-dependent DNA damage response and can reduce the recruitment or function of MDC1 and CHK2 at sites of DNA damage, attenuating signaling required for cell-cycle arrest and efficient DNA repair [37,38]. Tax has additionally been reported to interact with checkpoint proteins and to perturb ATR/CHK1 signaling, although the relative contribution of these effects appears to depend on the cellular context [38,39]. Furthermore, Tax can sequester several DNA damage response factors, including BRCA1, CHK2, DNA-PK, and MDC1, into Tax-containing nuclear structures, potentially reducing their availability at sites of DNA damage [8]. Together, these effects may allow HTLV-1-infected cells to continue proliferating despite persistent DNA lesions, thereby increasing the opportunity for accumulation of mutations and chromosomal abnormalities. Thus, the interaction between enhanced generation of replication-associated damage and impaired damage sensing, checkpoint activation, and repair may constitute a central feature of HTLV-1-driven genomic instability. Nevertheless, because the evidence for individual DNA repair defects varies across experimental systems, we focus here on TDP1 and MMR, for which the mechanistic links to nucleoside analog-induced replication stress are particularly relevant to the therapeutic framework discussed in this review.

## 7. SLFN11–TDP1 Axis as a Determinant of Replication Stress Tolerance and Therapeutic Vulnerability

The SLFN gene family was originally identified in murine thymocytes as regulators of lymphocyte development and cell-cycle quiescence, with the name “Schlafen” reflecting their growth-suppressive properties [40]. Comparative genomics later revealed mammalian expansion and species-specific diversification of SLFNs, consistent with roles in immune regulation and cellular stress responses.

SLFN11 was subsequently identified through unbiased pharmacogenomic analyses of human cancer cell lines as a strong predictor of hypersensitivity to DNA-targeting agents, including topoisomerase I inhibitors, platinum compounds, and nucleoside analogs, before its molecular function was defined [41,42,43,44,45]. Mechanistic studies later established SLFN11 as a replication stress execution factor rather than a DNA repair enzyme. SLFN11 is recruited to stressed replication forks through its association with RPA-coated single-stranded DNA (ssDNA) and enforces irreversible replication arrest by inhibiting fork progression and origin firing, thereby preventing tolerance and converting repairable stress into lethal replication failure [46]. Furthermore, SLFN11 inhibits RAD51-mediated protection of reversed replication forks, limiting fork stabilization and restart and thereby reinforcing replication stress-induced cytotoxicity [13].

Clinically, SLFN11 expression serves as a predictive biomarker across multiple tumor types [41,42,43,44,45,47]. Its frequent epigenetic silencing is associated with resistance to DNA-damaging therapies, whereas pharmacologic reactivation restores drug sensitivity in preclinical models, underscoring SLFN11 as a central regulator of replication stress tolerance and therapeutic response.

### 7.1. Evidence from CRISPR Screening: SLFN11 Governs Irinotecan (CPT-11) Sensitivity Independently of TDP1

A key study employed CRISPR knockout screens in isogenic TDP1-deficient cells to identify genes that modulate sensitivity to CPT-11 [17]. Strikingly, loss of SLFN11 emerged as a top resistance-conferring event even in the complete absence of TDP1, indicating that SLFN11 operates through a pathway that is genetically separable from 3’-end processing.

Mechanistically, CPT-11 induces Top1–DNA cleavage complexes that stall replication forks. While TDP1 normally resolves abortive Top1 lesions, the CRISPR data demonstrate that SLFN11 loss restores survival not by enhancing lesion repair but by permitting replication restart and tolerance. Thus, SLFN11 functions as a dominant execution factor that determines whether RS is lethal or survivable, regardless of the efficiency of lesion removal.

### 7.2. Conceptual Shift: From SLFN11–TDP1 Cooperation to Parallel Control of Fork Fate

These findings necessitate a conceptual revision of the SLFN11–TDP1 relationship. Rather than acting in a linear or epistatic pathway, SLFN11 and TDP1 control RS outcomes at orthogonal levels: • TDP1 determines whether obstructive DNA termini can be chemically resolved. • SLFN11 determines whether stressed replication forks are allowed to restart.

In this framework, TDP1 deficiency increases the burden of unresolved lesions, while SLFN11 expression determines whether this burden is tolerated or converted into irreversible fork arrest. Importantly, SLFN11 can impose lethality even when alternative repair or bypass pathways partially compensate for TDP1 loss.

### 7.3. Future Perspectives for Nucleoside Analog Sensitivity in ATL

A role for SLFN11 in abacavir and CPT-11 response applying this model to HTLV-1-mediated ATL suggests a revised interpretation of abacavir sensitivity. While prior studies established TDP1 suppression as a determinant of abacavir and CPT-11 cytotoxicity, Zhang et al. raise the possibility that SLFN11 status critically modulates this response [17].

Abacavir, a chain-terminating nucleoside analog, or CPT-11, an inhibitor of Top1, induces replication stress through aberrant incorporation and stalled elongation [11]. In TDP1-low ATL cells, such lesions accumulate; however, whether these lesions trigger cell death may depend on SLFN11-mediated enforcement of fork arrest. SLFN11-positive ATL cells are therefore predicted to be hypersensitive to abacavir or CPT-11, whereas SLFN11-silenced cells may tolerate TDP1-dependent lesions via fork restart and alternative tolerance pathways.

### 7.4. Therapeutic and Biomarker Implications

**Figure 6:**
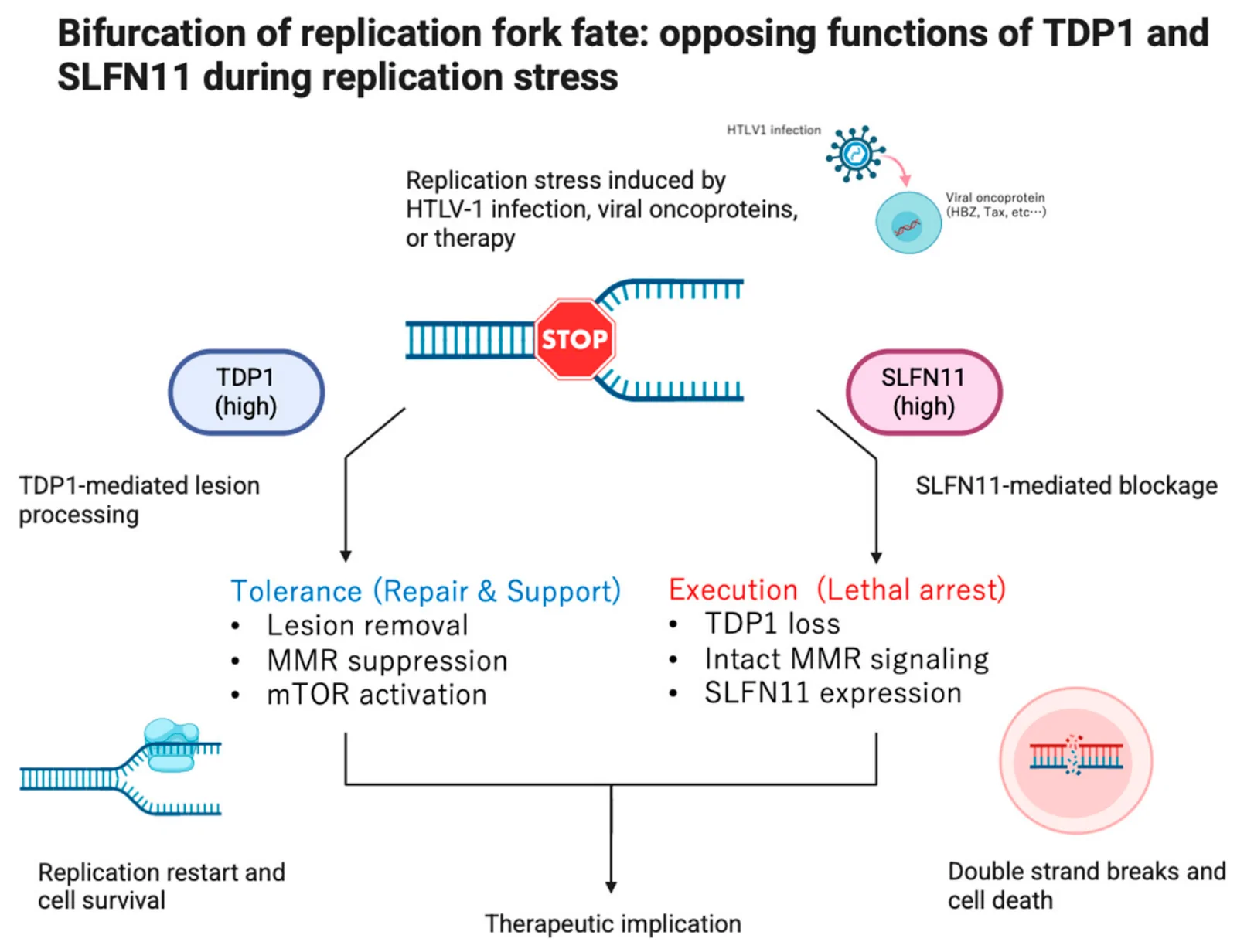
Bifurcation of replication fork fate: opposing functions of TDP1 and SLFN11 during replication stress. Replication stress generated by blocked 3′ DNA termini creates a critical branch point that determines replication fork fate. TDP1-mediated processing of blocked DNA ends removes replication-blocking lesions, allowing DNA synthesis to resume and promoting replication fork restart and cell survival. Conversely, SLFN11 enforces irreversible replication arrest by inhibiting replicative helicase progression and suppressing activation of dormant replication origins, thereby preventing fork recovery. Persistent fork arrest leads to replication fork collapse, accumulation of DNA double-strand breaks, and cell death. This functional opposition between TDP1-dependent lesion resolution and SLFN11-mediated fork restriction represents a central mechanism governing the balance between replication stress tolerance and lethal replication failure. This figure is created with BioRender.com.

The emerging picture positions SLFN11 as an independent and actionable determinant of RS-based therapies in ATL (Figure 6):

1) SLFN11 expression as a modifier of TDP1-based vulnerabilities Assessment of SLFN11 alongside TDP1 may better predict responses to abacavir and related nucleoside analogs than either marker alone.
2) Epigenetic reactivation strategies Restoring SLFN11 expression in TDP1-suppressed ATL cells is predicted to convert sublethal replication stress into irreversible cytotoxicity [43,48,49].
3) Combination approaches in SLFN11-negative / TDP1-deficient ATL, combining nucleoside analogs with ATR–CHK1 inhibition may overcome tolerance by disabling compensatory fork protection mechanisms.

Together, these implications support a model in which SLFN11 acts as a master switch that determines whether TDP1-associated replication stress is lethal or tolerated, providing a refined framework for therapeutic exploitation in ATL.

## 8. Outstanding Questions and Future Directions

- What is the full spectrum of nucleoside analog lesions processed by TDP1 versus other enzymes? A biochemistry-informed atlas would guide rational drug selection.
- How do MMR defects caused by viral proteins quantitatively alter sensitivity to different nucleoside analog chemotypes?
- Can small-molecule inhibitors of TLS polymerases or TDP1 modulators be safely combined with nucleoside analogs in the clinic?
- What are robust, clinically feasible biomarkers (IHC, mRNA, functional assays) to select patients for these strategies?

## 9. Conclusions

Replication stress (RS) has emerged as one of the defining vulnerabilities of malignant cells and an increasingly attractive therapeutic target. Rather than viewing the response to RS-inducing agents solely through the lens of DNA damage accumulation, this review proposes an integrated conceptual framework in which therapeutic outcome is determined by the dynamic balance between lesion processing and replication execution. Within this model, TDP1 and the mismatch repair (MMR) machinery function primarily as lesion-processing pathways that determine whether abnormal DNA termini or nucleotide misincorporation are repaired, bypassed, or converted into checkpoint-activating DNA lesions. In contrast, SLFN11 functions downstream as a replication execution factor that determines whether stalled replication forks are irreversibly arrested or allowed to restart. At the same time, mTOR signaling provides the metabolic and biosynthetic support required to sustain DNA synthesis under conditions of chronic RS, thereby influencing the threshold at which replication-associated damage becomes incompatible with cell survival. Together, these pathways form an interconnected RS tolerance network rather than acting as isolated determinants of drug response (Figure 1).

HTLV-1-associated adult T-cell leukemia/lymphoma (ATL) provides a particularly informative biological model for understanding this network. Unlike many malignancies in which replication stress tolerance evolves through the gradual accumulation of somatic alterations, ATL is driven by viral oncoproteins that coordinately reprogram host DNA damage responses. Viral suppression of TDP1 and MMR, together with persistent oncogene-driven replication stress and dependence on adaptive signaling pathways such as mTOR, generates a distinctive therapeutic landscape in which vulnerabilities arise not from a single defective repair pathway but from the collective rewiring of replication stress tolerance. The emerging evidence that SLFN11 independently determines the fate of replication forks further refines this model by demonstrating that the persistence of DNA lesions alone is insufficient to predict therapeutic response. Instead, lesion burden and replication execution represent complementary but genetically separable determinants of cytotoxicity (Figure 1).

This integrated framework has important translational implications. Biomarker-guided assessment of TDP1, MMR, SLFN11, and mTOR pathway activity may improve patient stratification for nucleoside analogs, topoisomerase I inhibitors, and emerging RS-targeted therapies. Furthermore, rational combination strategies that simultaneously increase replication-associated DNA damage while disabling adaptive tolerance mechanisms—including SLFN11-directed approaches, ATR–CHK1 inhibition, or metabolic interventions targeting mTOR signaling—may achieve greater therapeutic selectivity than conventional cytotoxic regimens. Such strategies aim not merely to increase DNA damage but to prevent cancer cells from adapting to persistent replication stress (Figure 2).

Although this review focuses on ATL, the conceptual framework described here is likely applicable to a broad range of replication stress-driven malignancies characterized by oncogene activation, defective DNA repair, or chronic therapeutic stress. As genomic, functional genomic, and pharmacologic datasets continue to expand, integrating lesion-processing pathways with replication execution mechanisms will facilitate the development of predictive biomarkers and mechanism-based therapeutic combinations. Ultimately, understanding how cancer cells balance DNA damage resolution with replication stress tolerance may provide a general blueprint for precision therapies that exploit one of the most fundamental liabilities of human cancer.

## Data Availability

The raw data supporting the conclusions of this article will be made available by the authors on request.

## Abbreviations

The following abbreviations are used in this manuscript:

RS	Replication Stress
HTLV-1	human T-cell leukemia virus type 1
ATL	adult T-cell leukemia/lymphoma
TDP1	tyrosyl-DNA phosphodiesterase 1
MMR	mismatch repair
CPT-11	irinotecan
SLFN11	Schlafen 11
CTNAs	Chain-terminating nucleoside analogs
BER	base excision repair
SSBs	single-strand breaks
TLS	translesion synthesis
HBZ	HTLV-1 bZIP factor
